# RETRACTION: Effect of Two Different Laparoscopic Techniques on Post‐Operative Wound Complications in Patients With Benign Gynaecological Diseases: A Meta‐Analysis

**DOI:** 10.1111/iwj.70347

**Published:** 2025-03-18

## Abstract

**RETRACTION:** HeH. , LiT. , CuiM. , JiangQ. , JiangF. , LiM. , and LiuY. , “Effect of Two Different Laparoscopic Techniques on Post‐Operative Wound Complications in Patients With Benign Gynaecological Diseases: A Meta‐Analysis,” International Wound Journal 21, no. 2 (2024): e14382, 10.1111/iwj.14382.PMC1082852237830298

The above article, published online on 13 October 2023, in Wiley Online Library (http://onlinelibrary.wiley.com/), has been retracted by agreement between the journal Editor in Chief, Professor Keith Harding; and John Wiley & Sons Ltd. Following an investigation by the publisher, all parties have concluded that this article was accepted solely on the basis of a compromised peer review process. The editors have therefore decided to retract the article. The authors did not respond to our notice regarding the retraction.

**RETRACTION:** H. He , T. Li , M. Cui , Q. Jiang , F. Jiang , M. Li , and Y. Liu , “Effect of Two Different Laparoscopic Techniques on Post‐Operative Wound Complications in Patients With Benign Gynaecological Diseases: A Meta‐Analysis,” International Wound Journal 21, no. 2 (2024): e14382, 10.1111/iwj.14382.PMC1082852237830298

The above article, published online on 13 October 2023, in Wiley Online Library (http://onlinelibrary.wiley.com/), has been retracted by agreement between the journal Editor in Chief, Professor Keith Harding; and John Wiley & Sons Ltd. Following an investigation by the publisher, all parties have concluded that this article was accepted solely on the basis of a compromised peer review process. The editors have therefore decided to retract the article. The authors did not respond to our notice regarding the retraction.

